# Identification of a Hidden Lesion in Meckel's Cave: A Diagnostic Odyssey of Unilateral Facial Pain in a Nonagenarian

**DOI:** 10.7759/cureus.111197

**Published:** 2026-06-20

**Authors:** Prakash Velmurugan, Sarath Kumar Mohan Kumar, Kiran Chandramohan, Mussammat Fairuj, Mirza Ziaul Haque

**Affiliations:** 1 Geriatric Medicine, Medway NHS Foundation Trust, Gillingham, GBR; 2 General Internal Medicine, Medway Maritime Hospital, Gillingham, GBR; 3 General Internal Medicine, Medway NHS Foundation Trust, Gillingham, GBR

**Keywords:** elderly patient, facial pain, fibrous dysplasia, meckel's cave, petrous apex, skull base lesion, trigeminal neuralgia

## Abstract

Fibrous dysplasia of the temporal bone and petrous apex is an uncommon benign fibro-osseous disorder, particularly in elderly individuals. Involvement of the skull base may present with atypical cranial neuropathies and may mimic inflammatory, vascular, or neoplastic diseases. This report describes a 90-year-old woman with recurrent unilateral facial pain and episodic facial muscle contractions. Initial assessment considered neoplastic pathology and inflammatory vasculopathy. Serial clinical evaluations, laboratory investigations, and neuroimaging established the diagnosis of left petrous apex fibrous dysplasia extending into Meckel's cave and the internal auditory meatus, resulting in mass effect on the trigeminal nerve. Multidisciplinary neurosurgical and neurological consultation advised conservative management due to advanced age, stable symptoms, absence of progressive neurological deficits, and favourable response to pregabalin. This case highlights a rare and diagnostically challenging skull base lesion with overlapping features of trigeminal neuralgia and hemifacial spasm and underscores the importance of avoiding invasive interventions in carefully selected frail elderly patients when symptom control is achievable through conservative measures.

## Introduction

Fibrous dysplasia (FD) is a nonheritable, benign skeletal disorder resulting from genetic mutations [1]. FD typically manifests in childhood, though the age of onset ranges from nine to 55 years, with equal sex distribution [2,3]. The condition may be monostotic or polyostotic, affecting one or multiple bones, and occurs throughout the skeleton with a predilection for long bones, ribs, and craniofacial structures [4]. FD results from mutations in the guanine nucleotide-binding protein, alpha-stimulating activity polypeptide 1 (GNAS1) gene on chromosome 20, which inhibit osteoblastic lineage differentiation and may lead to expansile lesions with compressive symptoms [1]. FD is considered rare, with an estimated incidence of 1:4,000-1:10,000 [5]. Within the skull base, the ethmoid bone is most commonly affected, followed by the sphenoid bone, frontal bone, and temporal bone, which accounts for only 24% of the skull base dysplasia [6].

Temporal bone FD accounts for a minority of craniofacial cases, most commonly presenting with headache (59%), followed by hearing loss (29%), with cranial neuropathy less frequent [7]. Meckel's cave, located within the temporal bone, is a cerebrospinal fluid-filled pouch in the middle cranial fossa containing the trigeminal nerve and ganglion [8]. In a study among 12 patients with mass lesions in Meckel's cave, most exhibited fifth cranial nerve symptoms, but only three reported facial pain [9].

Neuralgias are characterised by brief, lancinating pain; although most are primary, facial pain associated with cranial nerve symptoms or signs should prompt investigation for secondary causes and warrant imaging to identify underlying pathology [10]. Diagnostic uncertainty increases in elderly patients due to overlapping atypical presentations with more common vascular or neurological conditions. Magnetic resonance imaging (MRI) is the preferred imaging modality to assess Meckel's cave and its contents and adjacent structures [11]. Though histopathological findings along with imaging and clinical findings help in establishing a complete diagnosis, when obtaining a satisfactory biopsy is challenging, radiologic and clinical findings can provide a high probability of diagnosis [1].

A 90-year-old woman presented with recurrent, episodic unilateral facial pain and hemifacial spasms. Initial investigations focused on excluding inflammatory arteritis and malignancy. MRI revealed FD of the left petrous apex extending into Meckel's cave and compressing the trigeminal nerve. This case highlights the necessity of thorough diagnostic evaluation and consideration of rare aetiologies, including skull base fibro-osseous lesions, in elderly patients with atypical facial pain.

## Case presentation

A 90-year-old woman was referred for the assessment of recurrent, intermittent left-sided facial pain and headache of two months' duration. The pain was localised to the left temporal region and cheek, occurring four to five times daily, with each episode lasting approximately five minutes and resolving spontaneously. During these episodes, involuntary contraction of the left cheek, closure of the left eye, and elevation of the corner of the mouth were observed. The increasing frequency of these episodes gradually interfered with her daily functioning.

Her medical history included hypertension, transient ischaemic attack, congestive cardiac failure, hypothyroidism, and osteoporosis. She lived independently, supported by her children. She did not smoke and consumed alcohol occasionally.

Several months before the presentation, the patient visited an optician for the evaluation of left eye pain; the ocular examination was normal. She later presented to the emergency department with a persistent unilateral headache and left-sided facial discomfort. On initial assessment, cognition was preserved; cranial nerve examination was normal; limb power was intact; and no focal neurological deficits were detected. Her blood pressure was elevated at 180/94 mmHg.

She was referred to the Frailty Same-Day Emergency Care (SDEC) service for further evaluation of the left hemifacial pain. General examination, including the cardiovascular, respiratory, and abdominal systems, was unremarkable. Cranial nerve assessment demonstrated intact function of the cranial nerves II-XII. The patient had tenderness over the V1 (ophthalmic distribution) of the trigeminal nerve. Subsequent examination of the trigeminal nerve (cranial nerve V) showed symmetrical sensation to light touch and pinprick bilaterally across the ophthalmic (V1), maxillary (V2), and mandibular (V3) distributions. The muscles of mastication, including the masseter and temporalis, displayed normal bulk and power bilaterally. Jaw opening and side movements were normal, and corneal reflexes remained bilaterally present with normal jaw jerk.

In view of the temporal tenderness, an empirical course of prednisolone 60 mg daily for seven days was commenced while further investigations were undertaken to exclude giant cell arteritis. Routine blood tests, including inflammatory markers, were normal. Subsequent review in the Frailty SDEC clinic showed normal inflammatory markers, with C-reactive protein at 0.6 mg/L and an erythrocyte sedimentation rate within normal limits. There was no history of fever, jaw claudication, scalp tenderness, visual disturbance, weight loss, constitutional symptoms, or polymyalgia rheumatica-type symptoms. Repeat neurological examinations remained normal between symptomatic episodes. The initial mild left temporal tenderness resolved (Tables 1-5).

**Table 1:** Full blood count MCH: mean corpuscular haemoglobin; MCHC: mean corpuscular haemoglobin concentration; MCV: mean corpuscular volume; RDW: red blood cell distribution width; RBC: red blood cell; WBC: white blood cell

Parameter	Day 1	Day 30	Reference range
Haemoglobin	113	105	115-165 g/L
Haematocrit	0.34	0.32	0.37-0.47 L/L
MCH	27.6	26.5	27-32 pg
MCHC	330	325	320-360 g/L
MCV	83.7	81.4	80-100 fL
RDW	13	14	11.5-14.5%
RBC	4.1	3.97	3.8-5.8x10^12^/L
Platelet count	234	209	140-400x10^9^/L
WBC	7.0	4.6	3.6-11.0x10^9^/L

**Table 2:** Inflammatory markers ESR: erythrocyte sedimentation rate; CRP: C-reactive protein

Parameter	Day 1	Day 30	Reference range
ESR	5	6	0-20 mm/hr
CRP	0.4	0.6	<5 mg/L

**Table 3:** Renal function and electrolytes eGFR: estimated glomerular filtration rate; AKI: acute kidney injury

Parameter	Day 1	Day 30	Reference range
eGFR	71	64	>90 mL/min/1.73 m^2^
Creatinine	68	74	45-84 μmol/L
AKI	0	0	0
Sodium	133	134	135-146 mmol/L
Potassium	4.5	4.2	3.5-5.3 mmol/L
Adjusted calcium	2.45	2.37	2.1-2.6 mmol/L
Phosphate	1.10	1.06	0.8-1.5 mmol/L

**Table 4:** Liver function test

Parameter	Day 1	Day 30	Reference range
Total bilirubin	7	8	0-21 μmol/L
Alkaline phosphatase	54	51	30-130 IU/L
Alanine transaminase	12	11	10-40 IU/L
Albumin	40	39	35-50 g/L

**Table 5:** Endocrine investigations TSH: thyroid-stimulating hormone

Parameter	Day 1	Reference range
Free T4	13.1	12-22 pmol/L
TSH	3.03	0.27-4.5 mIU/L
Vitamin D	89	50 nmol/L

Despite a brief empirical course of corticosteroids, the patient continued to experience stereotyped episodes of facial tightening and unilateral pain. Alternative neurological diagnoses considered included trigeminal neuralgia, hemifacial spasm, cervical radiculopathy, facial nerve irritation, skull base pathology, and atypical migraine.

MRI of the brain, internal auditory meatus, and cervical spine demonstrated an expansile lesion involving the left petrous temporal bone. The lesion was heterogeneous on T2-weighted images and mildly hyperintense on T1-weighted images relative to grey matter. The lesion occluded the left internal auditory meatus and extended into Meckel's cave, resulting in a mass effect on the left trigeminal nerve. Partial encasement of the internal carotid artery was also observed. Additional findings included distortion of the bony and membranous labyrinth and fluid within the left mastoid air cells.

Additional imaging findings included mild diffuse cerebral atrophy and Fazekas grade 2 small-vessel ischaemic changes. Cervical spine imaging showed multilevel degenerative disc disease with mild foraminal narrowing, without significant spinal canal stenosis.

Figures 1-3 present MRI of the brain images demonstrating lesion extent and adjacent neural involvement.

**Figure 1:**
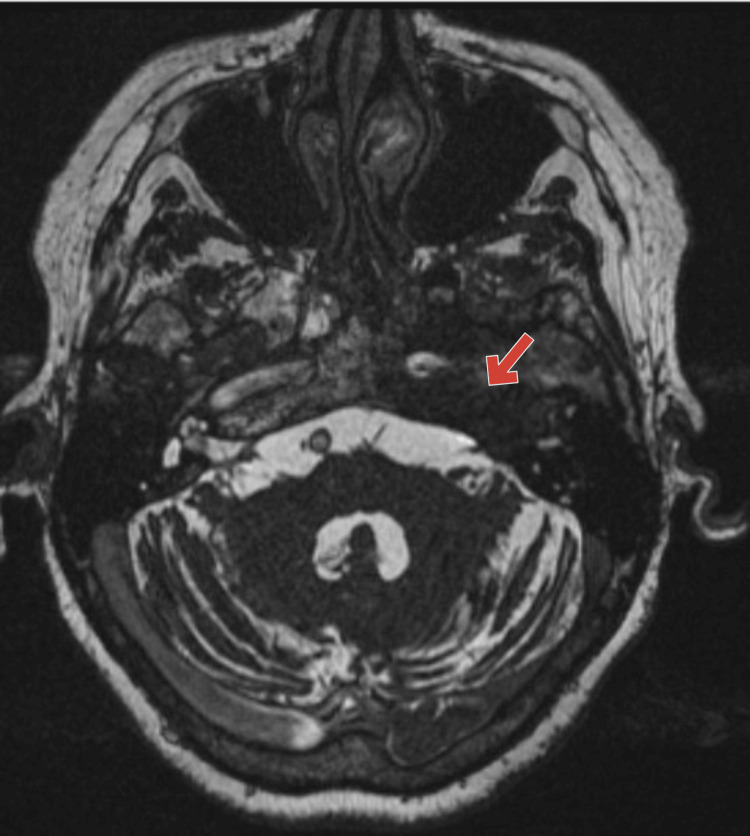
Post-contrast T1-weighted image This figure demonstrates an expansile enhancing lesion involving the left petrous apex and temporal bone. The lesion extends into Meckel's cave and the internal auditory meatus, producing mass effect on adjacent neural structures.

**Figure 2:**
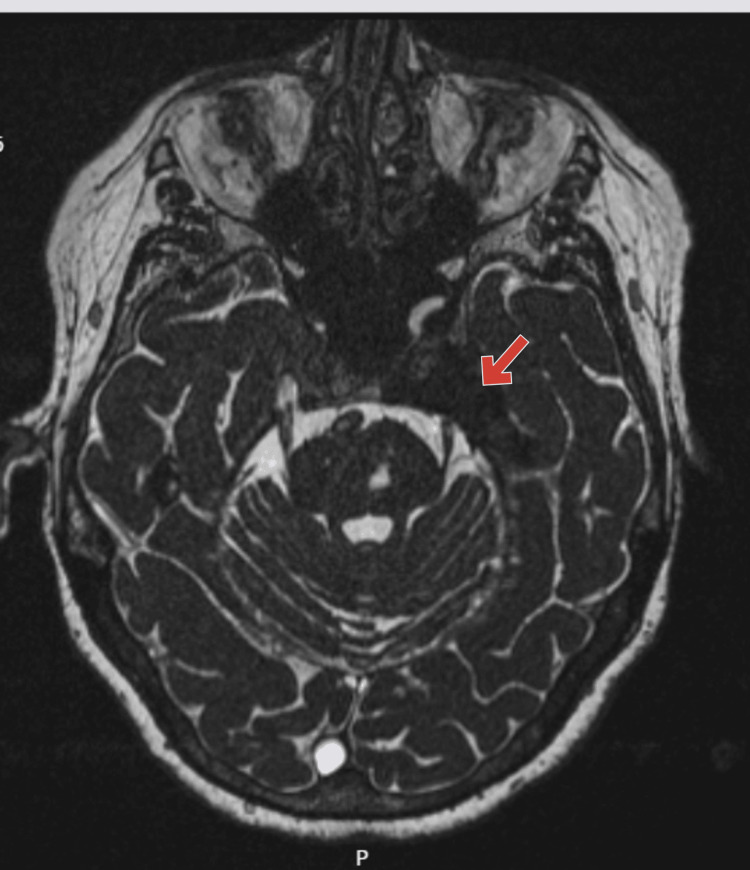
Non-contrast T2-weighted image This figure shows a heterogeneous lesion within the left petrous apex with mixed signal intensity. Associated distortion of adjacent skull base anatomy and extension toward Meckel's cave are evident.

**Figure 3:**
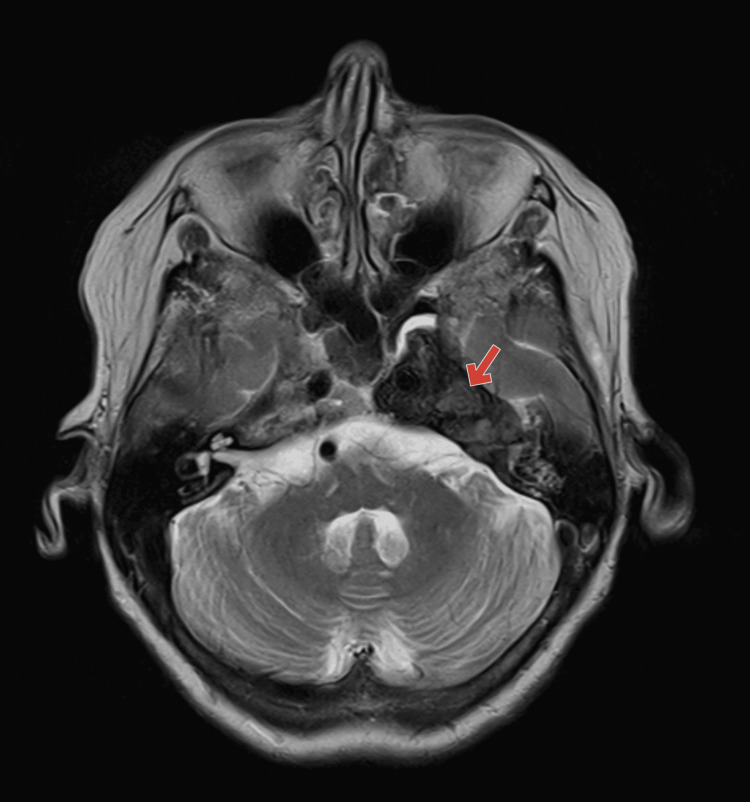
T2-weighted high-resolution image This figure provides detailed visualisation of the lesion's relationship to the trigeminal nerve within Meckel's cave. The image highlights compression of the nerve and involvement of the internal auditory canal and surrounding skull base structures.

Radiological evaluation supported a diagnosis of left temporal bone FD involving the petrous apex, Meckel's cave, and internal auditory meatus.

The patient's condition was discussed with neurology and tertiary neurosurgical teams. Neurosurgical assessment found no indication for urgent surgical intervention, as there was no evidence of significant brain compression. Considering her advanced age, cardiovascular comorbidities, stable episodic symptoms, and absence of significant functional decline, conservative management was recommended. Additional contrast imaging and computed tomography were reserved for situations in which further lesion characterisation was clinically warranted.

The patient's symptoms improved significantly with low-dose pregabalin 25 mg daily. Corticosteroids were discontinued when the likelihood of inflammatory arteritis was considered low. The patient was followed up at two visits. At the first visit, within two weeks, she reported reduced pain frequency and mild improvement in severity. At the second visit, four weeks after the initial assessment, she was completely symptom-free and had resumed daily activities. She remained neurologically stable, with no evidence of progressive cranial nerve deficits, hearing loss, or limb weakness.

## Discussion

Unilateral, episodic neuropathic pain is most commonly attributed to trigeminal neuralgia, yet temporomandibular disorders represent the most frequent non-dental cause of facial pain. In patients over 50 years of age, it is essential to exclude giant cell arteritis when considering temporomandibular aetiologies [12]. In this nonagenarian, recurrent, episodic unilateral facial pain in the trigeminal distribution prompted the initial consideration of trigeminal neuralgia. However, the patient's advanced age necessitated prioritisation of secondary and potentially serious causes, such as giant cell arteritis and malignancy, over autoimmune conditions, which are exceedingly rare in this demographic. Classical manifestations of giant cell arteritis include headache, jaw claudication, polymyalgia rheumatica, and visual symptoms, but up to 40% of patients may present atypically [13]. The absence of typical features, along with normal inflammatory markers (erythrocyte sedimentation rate (ESR) and C-reactive protein (CRP)), repeatedly obviated the need for temporal artery biopsy. High-resolution MRI of the head was subsequently performed to exclude malignancy and revealed FD involving Meckel's cave, which is not a common area for FD [6], accounting for the trigeminal neuralgia-like symptoms in this elderly patient and excluding malignancy.

Carbamazepine is the first-line therapy for trigeminal neuralgia [14], but our patient did not tolerate this medication. Pregabalin was initiated, given its demonstrated efficacy for paroxysmal facial pain in pilot studies [15], resulting in symptomatic improvement and sustained relief on follow-up. The case was discussed with a tertiary neurosurgical unit, which recommended continued conservative management as the patient was neurologically stable and responding to medical therapy.

This case underscores several important points: (1) FD should be considered in the differential diagnosis of atypical cranial neuropathies, even in elderly patients, and (2) diagnosis requires the careful integration of clinical, laboratory, and imaging findings. By expanding the clinical spectrum of skull base FD to include advanced age with concurrent trigeminal neuralgia-like pain and hemifacial spasm, this report provides valuable insights for clinicians managing similar presentations.

Limitations

This is a single-patient case report, limiting the generalisability of its findings. The association between petrous apex FD and the patient's trigeminal neuralgia-like pain is based on radiological evidence of trigeminal nerve compression and observed clinical response to neuropathic pain treatment; thus, a definitive causal relationship cannot be established.

Histopathological confirmation was not obtained, as biopsy or surgical intervention was not clinically indicated, given the patient's advanced age, characteristic radiological findings, and stable neurological status. While the patient experienced symptomatic improvement with pregabalin and remained neurologically stable during follow-up, the long-term natural history of the lesion, risk of progression, and durability of symptom control could not be assessed.

## Conclusions

Petrous apex FD involving Meckel's cave is a rare aetiology for unilateral facial pain and cranial neuropathic symptoms. In this case, the lesion produced a unique combination of trigeminal neuralgia-like pain and episodic hemifacial spasm, initially mimicking neoplastic and inflammatory conditions. Repeated clinical assessments and MRI enabled diagnosis while avoiding unnecessary invasive interventions. Conservative management with neuropathic analgesia provided effective symptom control. This case highlights the importance of considering skull base fibro-osseous lesions in elderly patients presenting with atypical unilateral facial pain and cranial nerve symptoms.

## References

[1] Ricalde P, Magliocca KR, Lee JS (2012). Craniofacial fibrous dysplasia. Oral Maxillofac Surg Clin North Am.

[2] Wu H, Yang L, Li S (2014). Clinical characteristics of craniomaxillofacial fibrous dysplasia. J Craniomaxillofac Surg.

[3] Hanifi B, Samil KS, Yasar C, Cengiz C, Ercan A, Ramazan D (2013). Craniofacial fibrous dysplasia. Clin Imaging.

[4] DiCaprio MR, Enneking WF (2005). Fibrous dysplasia. Pathophysiology, evaluation, and treatment. J Bone Joint Surg Am.

[5] Menon S, Venkatswamy S, Ramu V, Banu K, Ehtaih S, Kashyap VM (2013). Craniofacial fibrous dysplasia: surgery and literature review. Ann Maxillofac Surg.

[6] Lustig LR, Holliday MJ, McCarthy EF, Nager GT (2001). Fibrous dysplasia involving the skull base and temporal bone. Arch Otolaryngol Head Neck Surg.

[7] Frisch CD, Carlson ML, Kahue CN (2015). Fibrous dysplasia of the temporal bone: a review of 66 cases. Laryngoscope.

[8] Battal B, Zamora C (2025). Imaging of Meckel cave: a pictorial review of anatomy and pathology. Roentgen Ray Rev.

[9] Beck DW, Menezes AH (1987). Lesions in Meckel's cave: variable presentation and pathology. J Neurosurg.

[10] Siccoli MM, Bassetti CL, Sándor PS (2006). Facial pain: clinical differential diagnosis. Lancet Neurol.

[11] Malhotra A, Tu L, Kalra VB (2018). Neuroimaging of Meckel's cave in normal and disease conditions. Insights Imaging.

[12] Zakrzewska JM (2013). Differential diagnosis of facial pain and guidelines for management. Br J Anaesth.

[13] Levine SM, Hellmann DB (2002). Giant cell arteritis. Curr Opin Rheumatol.

[14] Maarbjerg S, Di Stefano G, Bendtsen L, Cruccu G (2017). Trigeminal neuralgia - diagnosis and treatment. Cephalalgia.

[15] Di Stefano G, Maarbjerg S, Truini A (2019). Trigeminal neuralgia secondary to multiple sclerosis: from the clinical picture to the treatment options. J Headache Pain.

